# Long-Term Assessment of Pelvic Organ Prolapse Reoperation Risk in Obese Women: Vaginal and Laparoscopic Approaches

**DOI:** 10.3390/jcm11226867

**Published:** 2022-11-21

**Authors:** Marine Lallemant, Géraldine Giraudet, Victoire Delporte, Hélène Behal, Chrystele Rubod, Sophie Delplanque, Yohan Kerbage, Michel Cosson

**Affiliations:** 1 Department of Gynecologic Surgery, Jeanne de Flandre University Hospital, 59000 Lille, France; 2 Santé Publique: Epidémiologie et Qualité des Soins, Unité de Biostatistiques, University of Lille, France CHU Lille, EA 2694, 59000 Lille, France

**Keywords:** obesity, POP repair, pelvic organ prolapse, recurrence, complications, reoperation, long-term outcomes

## Abstract

The aim of this study was to compare reoperation risks after pelvic organ prolapse repair at 5-year follow-up between obese, overweight, and normal-weight women and to assess these risks accounting for the surgical procedure. We performed a retrospective chart review of all the women who underwent POP repair by transvaginal mesh surgery between January 2005 and January 2009 or laparoscopic sacrocolpopexy between January 2003 and December 2013 at the Gynecologic Surgery Department of the Lille University Hospital. During the study period, 744 women who underwent POP repair were divided into three groups: 382 (51%), 240 (32%), and 122 (16%) in the nonobese group (BMI < 25 kg/m²), overweight group (25 kg/m² ≤ BMI < 30 kg/m²), and obese group (BMI ≥ 30 kg/m²), respectively. The primary outcome was global reoperation. The median duration of follow-up was 87 months. The risks of global reoperation did not significantly differ between the three BMI groups (adjusted HR (95% CI): 1.12 (0.69 to 1.82) for overweight women and 0.90 (0.46 to 1.74) for obese women compared to normal-weight women, adjusted *p* = 0.80), nor among the women who underwent transvaginal mesh surgery or laparoscopic sacrocolpopexy. The risks of reoperation for POP recurrence, stress urinary incontinence, or mesh-related complications did not significantly differ between the three BMI groups in the overall population nor accounting for the surgical procedure. In conclusion, obesity does not seem to be a risk factor of reoperation for POP recurrence, SUI, or mesh-related complications in the long term regardless of the surgical approach.

## 1. Introduction

Obesity is a medical condition in which an excess of body fat has accumulated. The body mass index (BMI) obtained by dividing the person’s weight (in kilograms) by the square of the person’s height (in meters squared) is a screening method for weight categories. For adults, a BMI between 25.0 and 29.9 kg/m² is defined as overweight; a BMI ≥ 30 kg/m² is defined as obese [1]. In the United States, current estimates are that 69% of adults are either overweight or obese, with approximately 35% being obese [2]. In France, 44% of women are overweight or obese [3]. The prevalence increases with age.

Obesity is a well-known risk factor for stress urinary incontinence (SUI) [4]. However, its role in pelvic organ prolapse (POP) remains controversial [5,6]. It is not a contraindication to prolapse surgery, whatever the surgical procedure [7,8]. Obesity is generally considered to be associated with an increased risk of perioperative and postoperative complications [9,10,11]. However, in the study by Thubert et al., the reoperation rate for recurrence 2 months after laparoscopic sacrocolpopexy in obese women was 2.5% [7]. In the literature, no study has yet assessed the risk of reoperation during long-term follow-up in obese women compared with non-obese women after prolapse repair regardless of the surgical procedure.

The main objective of this study was to compare the global reoperation rate at 5 years between obese, overweight, and normal-weight women regardless of the surgical approach and accounting for the type of surgical procedure. The secondary objective was to compare the risks of reoperation for recurrent prolapse, urinary incontinence (UI), and for mesh-related complications.

## 2. Materials and Methods

### 2.1. Study Design

We performed a retrospective chart review of all the women who underwent POP repair by transvaginal mesh surgery between January 2005 and January 2009 or laparoscopic sacrocolpopexy (LSCP) between January 2003 and December 2013 at the Gynecologic Surgery Department of the Lille University Hospital. The exclusion criterion was missing data about the BMI. The patients were divided into three groups: nonobese group (BMI < 25 kg/m²), overweight group (25 kg/m² ≤ BMI < 30 kg/m²), and obese group (BMI ≥ 30 kg/m²). The study protocol was approved by the institutional review board of the French College of Obstetricians and Gynecologists (#CEROG-2011-GYN-02-01 and #CEROG-2014-GYN-0903).

### 2.2. Surgical Procedures

Transvaginal Prolift^®^ mesh repair technique was standardized and described by Debodinance et al. [12]. An anterior and/or a posterior mesh was applied accounting for the prolapse type (anterior POP, posterior POP, or complete floor) [13]. The anterior mesh was inserted between the bladder and the vagina and fixed laterally by four arms passing through the obturator foramen, near the tendinous arch of the pelvic fascia. The posterior mesh was placed between the rectum and the vagina with an arm passing through the ischiorectal fossa and fixed in the sacrospinous ligament. The mesh was made of nonabsorbable polypropylene monofilament fibers (Prolift Pelvic Floor Repair System; Ethicon Women’s Health and Urology, Somerville, NJ, USA). Concomitant surgery was performed if necessary, such as a vaginal hysterectomy or traditional POP repair, including posterior sacrospinous fixation or colporrhaphy. In case of preexisting or occult SUI, a concomitant tension-free vaginal tape–obturator (TVT–O) sling was inserted according to the De Leval technique [14].

The LSCP procedure was performed as described by Wattiez and Cosson [15,16]. Pneumoperitoneum was established with a Veress needle. A 10 mm umbilical trocar was placed for the laparoscope. Two 5 mm right and left iliac trocars and one 10 mm suprapubic trocar were inserted. The surgeon was placed on the left side of the patient, his first assistant—on the right. Firstly, additional procedures such as subtotal or total hysterectomy and/or adnexectomy were performed. Then, the peritoneum overlying the sacrum was opened and the anterior longitudinal sacral ligament was exposed. Vaginal fornices were dissected by mobilizing the bladder anteriorly and the rectum posteriorly, thanks to a curved metal vaginal manipulator. Anteriorly, dissection reached the bladder trigone, and posteriorly, the levator ani muscles were exposed. Anterior and posterior meshes were sutured to the vaginal wall using absorbable sutures, with digital control to avoid transfixing the vagina and to control the level of dissection. In case of uterine conservation, the anterior mesh was inserted through the right broad ligament before reaching the promontory. One or both tails of the mesh were attached to the anterior sacral ligament by two permanent sutures. Meshes were peritonized to avoid small bowel obstruction. A concomitant treatment of urinary stress incontinence with a transobturator midurethral sling (MUS) was performed if stress urinary incontinence (SUI) caused by urethral hypermobility or occult SUI was diagnosed.

### 2.3. Data Collection

Analyzed data were obtained from two published studies that were conducted in our unit [13,17]. The data were collected from the hospital’s electronic medical records. Women were also called to limit misinterpretation of the reoperation rate. The women who underwent LSCP were contacted in November and December 2014 [17]. The women who underwent vaginal prolapse surgery [13] were contacted between August and October 2015 in order to have a similar follow-up. Noncontactable or nonconsenting patients were excluded from the data analysis.

Demographic and medical data and histories were collected during the preoperative consultation. A physical examination was performed to determine pelvic floor disorders. POP was classified according to the international POP-Q classification [18]. Stress urinary incontinence (SUI) was assessed by an interrogatory and a cough test before and after prolapse reduction with a speculum to search for occult urinary incontinence. Urodynamic exploration was performed in case of associated urinary incontinence. In cases of severe constipation and/or dyschesia, gastroenterological investigations, including defecography and anorectal manometry, were also performed. Dynamic pelvic MRI was prescribed depending on the case. POP surgeries were only performed if women had symptomatic POP with a POP-Q stage ≥ 2. Significant intraoperative complications (organ injuries and hemorrhage), early postoperative complications (hemorrhage, infection, or reoperation) were noted. All the patients underwent a physical examination two months after the surgery and remotely depending on the results of the clinical examination. Thereafter, the patients were seen again only in case of a new symptomatology appearance or persistent symptoms or a recurrence. POP recurrence was defined as a prolapse occurrence with a POP-Q stage ≥ 2 in at least one vaginal compartment.

If the woman told us during the phone call that she had undergone any new surgery at other hospitals, operative reports were collected and analyzed in order to obtain precise indications and nature of each reoperation. Reoperation data were extracted from both medical records (local and other hospitals) and call information.

### 2.4. Outcome Criteria

The primary outcome was global reoperation defined by reoperation for any indication.

Secondary outcomes were reoperation for recurrent prolapse, reoperation for SUI, and reoperation for mesh-related complications.

### 2.5. Statistics

Categorical variables were expressed as a number (percentage). Quantitative variables were expressed as the means and standard deviations or the medians and interquartile range. The normality of the distributions was checked graphically and with the Shapiro–Wilk test. The three BMI groups were compared using the baseline parameters and perioperative parameters with the chi-squared test or Fisher’s exact test for categorical variables and using analysis of variance for quantitative variables. Post hoc analyses were performed when significant results were found and the Bonferroni correction was applied. The cumulative incidence of global reoperation was estimated using the Kaplan–Meier method. The risk of global reoperation was compared between the three groups using the Cox proportional hazards regression model. To determine whether the surgical approach had an impact on the risk of reoperation, the interaction between the surgical approach and the three BMI groups was tested using the Cox model. The follow-up was censored at 5 years. The incidence of prolapse recurrence, reoperation for UI, and mesh-related complications was described and compared between the three groups using the same methods. Because of the low number of instances of prolapse recurrence, reoperation for UI and for mesh-related complications, adjustment was made for the predefined confounding factors only for the analysis of the primary outcome (global reoperation). The predefined confounding factors were age, parity, history of previous prolapse repair, cystocele stage, apical prolapse stage, rectocele stage, SUI surgery, concomitant hysterectomy, and the set-up of one or two meshes. Statistical tests were performed at the two-tailed α-level of 0.05. The data were analyzed using the SAS software package, release 9.4 (SAS Institute, Cary, NC, USA).

## 3. Results

**Figure 1:**
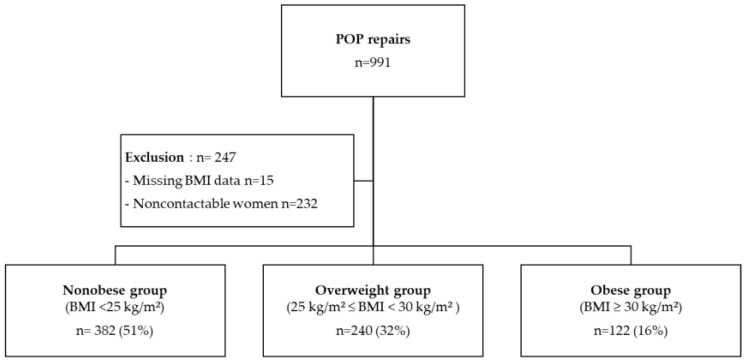
Flowchart.

During the study period, 991 POP repairs were performed (Figure 1). Two hundred and forty-seven women were excluded: 6% (15/247)—for missing BMI data, 94% (232/247)—for noncontactable women. Therefore, the analysis was based on 744 women: 386 (52%) and 358 (48%) underwent LSCP and transvaginal mesh surgery, respectively. The women were divided into three groups: 382 (51%), 240 (32%), and 122 (16%) in the nonobese, overweight, and obese groups, respectively. The median follow-up duration was 87 months.

In the overall study population, the mean age was 57.7 ± 10.8 years old (Table 1). Concerning the preoperative prolapse stage, 555 patients (74.6%) had a stage > 2 cystocele, 422 (56.7%) had a stage > 2 apical prolapse, and 312 (41.9%) had a stage > 2 rectocele.

Comparing the women according to their BMI group, there was no significant difference regarding their age (*p* = 0.46) or parity (*p* = 0.075). Regarding their surgical history, the overweight women had a more frequent history of hysterectomy than the nonobese women (22.5% vs. 14.4%; *p* = 0.029). There was no significant difference between the three groups in terms of previous prolapse repair (12.8% vs. 19.2% vs. 16.4%; *p* = 0.099) or SUI surgery (10.7 % vs. 13.3% vs. 13.9%; *p* = 0.50). The prolapse stage distribution was different between the three groups. The stage III–IV cystoceles were less frequent in the obese group compared with the nonobese group (58.2% vs. 74.9%, *p* < 0.001) and the overweight group (58.2% vs. 75.7%, *p* < 0.001). The stage III–IV apical prolapse rate was significantly lower in the obese group compared with the nonobese group (45.9% vs. 62.5%; *p* = 0.010) and in the overweight group compared to the nonobese group (53.3% vs. 62.6%, *p* = 0.042). The rectocele rates were comparable between the three groups (44.6% vs. 40.2% vs. 38.5%; *p* = 0.36). The women in the obese group underwent significantly more instances of transvaginal mesh surgery compared with the nonobese group (61.5% vs. 42.4%, *p* < 0.001) and the overweight group (61.5 vs. 50.4%, *p* < 0.001). The concomitant hysterectomy rate was similar between the three groups (*p* = 0.14). The concomitant SUI surgery rate was significantly lower in the nonobese group compared with the obese group (26.7% vs. 41%, *p* = 0.03) and the overweight group (26.7% vs. 36.7%, *p* = 0.03). There was no significant difference in terms of intraoperative complications between the three groups (*p* = 0.72).

**Figure 2:**
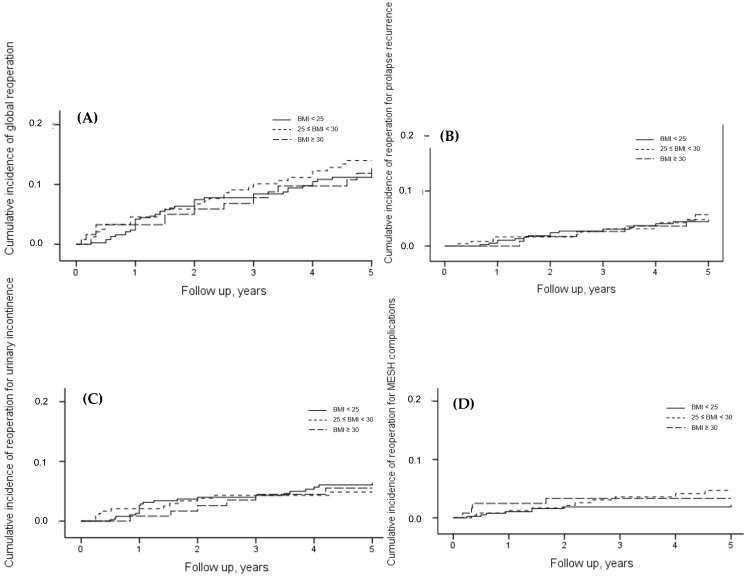
Cumulative incidence over 5 years of global reoperation, reoperation for prolapse recurrence, urinary incontinence, and mesh-related complications. (**A**) Cumulative incidence over 5 years of global reoperation. (**B**) Cumulative incidence over 5 years of prolapse recurrence. (**C**) Cumulative incidence over 5 years of reoperation for urinary incontinence. (**D**) Cumulative incidence over 5 years of reoperation for mesh-related complications. BMI: body mass index (kg/m^2^).

At 5-year follow-up, the rate of global reoperation was 13.0% (86/744). The risk of global reoperation did not significantly differ between the three groups of BMI (considering normal BMI as reference, the hazard ratios (95% CI) were 1.12 (0.70 to 1.78) for the BMI between 25 and 30 and 0.92 (0.49 to 1.72) for the BMI ≥ 30, global *p*-value = 0.83) even after adjustment for the predefined confounding factors (*p* = 0.80) (Figure 2 and Table 2). There was no interaction between the surgical procedure and the BMI in terms of global reoperation (*p*-value of the interaction with adjustment = 0.61): the risk of global reoperation did not significantly differ between the three groups of BMI whether it was for the women who underwent transvaginal mesh surgery (adjusted HR (95% CI): 1.49 (0.70 to 3.15) for the BMI between 25 and 30 and 1.05 (0.42 to 2.60) for the BMI ≥ 30, global adjusted *p*-value = 0.66) or laparoscopic sacrocolpopexy (adjusted HR (95% CI): 0.91 (0.47 to 1.74) for the BMI between 25 and 30 and 0.91 (0.34 to 2.38) for the BMI ≥ 30, global adjusted *p*-value = 0.99) (see Table 2).

At 5-year follow-up, 5.0% (32/744), 5.8% (39/744), and 3.2% (22/744) of the women underwent a reoperation for prolapse recurrence, SUI, and mesh-related complications, respectively. Among the 22 women who underwent a reoperation for a mesh-related complication, 81.8% (18/22) had mesh exposure, 13.6% (3/22)—mesh retraction, 4.5% (1/22)—mesh infection. The risks of reoperation for prolapse recurrence, SUI, and mesh-related complications did not significantly differ between the three BMI groups in the overall population (Table 2, Table 3 and Figure 2): the risk of reoperation for prolapse recurrence (the HRs (95% CI) were 0.99 (0.44 to 2.18) for the BMI between 25 and 30 and 1.13 (0.44 to 2.89) for the BMI ≥ 30, global *p*-value = 0.96); the risk of reoperation for SUI (the HRs (95% CI) were 0.79 (0.38 to 1.64) for the BMI between 25 and 30 and 0.83 (0.33 to 2.05) for the BMI ≥ 30, global *p*-value = 0.80); the risk of reoperation for mesh-related complications (the HRs (95% CI) were 1.98 (0.78 to 5.02) for the BMI between 25 and 30 and 1.56 (0.47 to 5.20) for the BMI ≥ 30, global *p*-value = 0.35). Regardless of the surgical procedure, there were no significant differences between the three BMI groups in terms of reoperation for prolapse recurrence, SUI, and mesh-related complications (*p*-value of interaction > 0.05) (see details in Table 2).

## 4. Discussion

In our study, 51% and 48% of the women were nonobese and overweight or obese, respectively. In the French ESTEBAN cohort, the rate of overweight or obese women was similar (44%) [3].

In the literature, the highest risk factors for the development of POP are age, parity, and vaginal delivery [19]. The association between obesity and POP seems to be more controversial [6]. Giri et al. published a meta-analysis suggesting an association between obesity and POP. However, they were unable to confirm it due to the lack of prospective studies and understanding of the mechanisms [5]. Moreover, this meta-analysis showed that this association was mostly valid for anatomic prolapse and not so much for symptomatic prolapse. This fact could explain the heterogeneity of results in the literature regarding the impact of obesity on pelvic organ prolapse. In our study population, and therefore in the symptomatic patients, cystoceles and apical prolapses in the obese patients were of a lower stage according to the POP-Q classification. There was no difference in terms of rectoceles. In the study of Washington et al., obesity had the same impact on all three vaginal compartments [20].

The reoperation rates for POP recurrence in our study population ranged from 4.8% to 5.7% depending on the BMI (unadjusted analysis). This rate is lower than the 9.5% rate of reoperation for surgical failure at 5 years in the study of Clark et al. [21]. However, they evaluated a cohort of women defined in 1995. Surgical techniques have evolved since then. However, the study was consistent with the 4.4% rate in the patients who underwent LSCP in the follow-up interval of 6 months to 3 years [22] and the 5% rate of reoperations for recurrence over the 5-year follow-up period in the patients who underwent transvaginal mesh surgery [23]. In the recent study by Shah et al., the reoperation rate for POP recurrence after apical suspension with a maximum follow-up of 14.8 years was 5.1% [24].

Long-term reoperation rates for POP recurrence were similar between the three BMI groups and were independent of the surgical approach (unadjusted analysis). The mean follow-up period was 87 months. Similarly to the studies by Thubert et al. [7], Bradley et al. [25], and Rappa et al. [26], there was no difference in prolapse recurrence (reoperated or not) in the obese and nonobese patients in the first year or two after POP repair regardless of the procedure: laparotomy, laparoscopic or vaginal procedure. However, these studies only reported short- or medium-term outcomes.

In our study, the obese patients were more often operated using the vaginal approach than the nonobese patients (61.5% vs. 42.4%, *p* = 0.001) or overweight patients (61.5% vs. 50.4%, *p* = 0.001). Thubert et al. showed the technical feasibility of LSCP in obese patients [7]. Nevertheless, laparoscopy in gynecologic surgery may provide some difficulties in obese patients. As described by Afors et al., the Trendelenburg position and pneumoperitoneum increase the intraabdominal pressure and induce a ventilation perfusion mismatch with potential hypoxemia [27]. These two laparoscopic requirements are essential to succeed in performing LSCP by exposing the sacral promontory. In our study, the inability to obtain these conditions probably deterred surgeons from performing LSCP in the obese patients. Furthermore, prospective studies comparing laparoscopic and vaginal POP repair demonstrated that operative time was significantly longer with the laparoscopic approach [28,29]. In addition, higher risks of nerve injuries as a result of prolonged compression and pressure sores in obese patients can also dissuade surgeons from performing this procedure. Even if laparoscopy in obese patients is feasible and confers advantages such as shorter hospital stay, less post-operative pain, and fewer wound infections without increasing complications [27,30,31], these challenging requirements might have led our surgical team to preferentially proposing vaginal POP repair in the obese patients. Robot-assisted laparoscopy could be an interesting alternative. Indeed, studies have demonstrated its safety compared with laparoscopy without robotic assistance in obese patients [32,33,34,35,36,37]. In the study by Joubert et al., complication rates and outcomes were similar in the obese women who underwent laparoscopic sacrocolpopexy and robot-assisted laparoscopic sacrocolpopexy [37]. Further studies are necessary to clarify the relevance of robotic surgery for POP repair in obese patients.

In terms of functional or anatomic success, the surgical approach to POP repair remains a matter of real debate regardless of the BMI. Maher et al. performed a randomized clinical trial comparing LSCP with transvaginal mesh repair (Prolift^®^,Gynecare, Ethicon Inc, Johnson and Johnson) in apical prolapse [28]. This study demonstrated that LSCP was associated with a higher anatomic success at 2-year follow-up, less intraoperative bleeding, a shorter duration of hospitalization, and a quicker return to the activities of daily living. Only the operative time was longer. Conversely, in a randomized clinical trial study (PROSPERE study) comparing LSCP and transvaginal mesh surgery for cystocele repair, LCSP offered equivalent success rates to vaginal meshes [29]. However, it was safer and featured a lower rate of complications and reoperations and a better preservation of the sexual function. Our retrospective study suggested that there was no difference between the two approaches in the obese patients.

Our study is original in comparing long-terms outcomes of POP repair according to the BMI. The mean follow-up time was 87 months. To our knowledge, this follow-up period is the longest described in literature. The surgeries were performed at a single center, but by an experienced team guaranteeing relative homogeneity of the procedures. The main limitation of this study is its retrospective design and lack of randomization. Retrospective collection of the baseline data generally misestimates adverse events and introduces measurement bias. The patients were contacted in order to limit the misinterpretation of the reoperation rate. However, the rate of women excluded because they were not contactable was moderately high (23%). The explanation is that the contact information was invalid, probably because of a long interval between the surgery and the call. We chose to divide the patients into three groups: nonobese, overweight, and obese. The severity of a patient’s obesity plays an important clinical role in the baseline characteristics, surgical approaches, and clinical outcomes. In our study, we could not assess surgical outcomes according to the severity of the obesity (BMI of 30–35 kg/m², 35–40 kg/m², and BMI > 40 kg/m²) because the BMI > 35 kg/m² cases were not sufficient for their evaluation. Furthermore, because of the low number of prolapse recurrences, reoperations for UI and for mesh-related complications, adjustment was made for the predefined confounding factors only for the analysis of the global reoperation rate. Interpretation of prolapse recurrence, reoperation for UI and for mesh-related complications rates should take this into account. Plus, we did not compare pelvic organ prolapse symptoms according to the BMI because validated questionnaires were not use during this study period.

## 5. Conclusions

In our study, obesity did not appear to be a risk factor of reoperations for POP recurrence, SUI, or mesh-related complications regardless of the surgical approach. The impact of obesity on the stages of POP and the vaginal compartments involved in the POP remains controversial. Further studies are necessary in order to determine the best surgical approach for this category of patients.

## Figures and Tables

**Table 1:** Comparison of the baseline and surgical characteristics and intraoperative complications between the nonobese, overweight, and obese groups.

	Total n = 744	Nonobese Group (BMI < 25 kg/m²) n = 382	Overweight Group (25 ≤ BMI < 30 kg/m²) n = 240	Obese Group (BMI ≥ 30 kg/m²) n = 122	*p*
BMI (kg/m²), mean ± SD	25.7 ± 4.4	22.4 ± 1.74	27.1 ± 1.4	32.8 ± 2.8	–
Age (years), mean ± SD	57.7 ± 10.8	57.6 ± 11.3	58.4 ± 10.2	56.9 ± 10.6	0.46
Parity, median (IQR)	2 (2–3)	2 (2–3)	3 (2–3)	3 (1–3)	0.075
Surgical history					
Prolapse surgery	115 (15.5)	49 (12.8)	46 (19.2)	20 (16.4)	0.099
Hysterectomy	130 (17.5)	55 (14.4)	54 (22.5)	21 (17.2)	0.035 *
SUI surgery	90 (12.1)	41 (10.7)	32 (13.3)	17 (13.9)	*0.50*
POP-Q stage					
Cystocele					*<0.001 ^†,§^*
0–I	129 (17.4)	54 (14.2)	39 (16.3)	36 (29.5)	
II	57 (7.7)	23 (6.1)	19 (7.9)	15 (12.3)	
III–IV	555 (74.9)	303 (79.7)	181 (75.7)	122 (58.2)	0.004 ^†,^*
Apical prolapse					
0–I	216 (29.1)	89 (23.4)	82 (34.2)	45 (36.9)	
II	104 (14.0)	53 (13.9)	30 (12.5)	21 (17.2)	
III–IV	422 (56.9)	238 (62.6)	128 (53.3)	56 (45.9)	
Rectocele					*0.36*
0–I	250 (33.8)	126 (33.3)	86 (36.0)	38 (31.2)	
II	178 (24.0)	84 (22.2)	57 (23.8)	37 (30.3)	
III–IV	312 (42.2)	169 (44.6)	96 (40.2)	47 (38.5)	
Surgical approach					*0.001 ^†,§^*
Sacrocolpopexy	386 (51.9)	220 (57.6)	119 (49.6)	47 (38.5)	
Transvaginal surgery	358 (48.1)	162 (42.4)	121 (50.4)	75 (61.5)	
Concomitant surgery					
Hysterectomy	273 (36.7)	148 (38.7)	76 (31.7)	49 (40.2)	*0.14*
SUI	240 (32.3)	102 (26.7)	88 (36.7)	50 (41.0)	0.003 ^†,^*
Intraoperative complications	23 (3.1)	10 (2.6)	9 (3.8)	4 (3.3)	*0.72*

The values are expressed as the number of cases (%) unless otherwise indicated. BMI: body mass index; SD: standard deviation; IQR: interquartile range; SUI: stress urinary incontinence; POP-QS: pelvic organ prolapse quantification. ^†^ Significant comparison between the nonobese group (BMI < 25 kg/m²) and the obese group (BMI ≥ 30). ^§^ Significant comparison between the overweight group (25 < BMI < 30 kg/m²) and the obese group (BMI ≥ 30 kg/m²). * Significant comparison between the nonobese group (BMI < 25 kg/m²) and the overweight group (25 < BMI < 30 kg/m²).

**Table 2:** Comparisons between the three BMI groups according to the initial surgical approach of global reoperation.

Global Reoperation								
Surgical Approach	BMI (kg/m²)	Number of Events	Five-Year Rates	Hazard Ratio (95% CI)	*p*	Adjusted Hazard Ratio (95% CI)	Adjusted *p*	Interaction of the *p* with the Adjustment
	<25	43/382	12.7%	1.00 (ref.)	0.83	1.00 (ref.)	0.80	
All	25–30	30/240	14.0%	1.12 (0.70 to 1.78)		1.12 (0.69 to 1.82)		–
	≥30	13/122	11.9%	0.92 (0.49 to 1.72)		0.90 (0.46 to 1.74)		
Transvaginal mesh surgery	<25	14/162	8.6%	1.00 (ref.)	0.60	1.00 (ref.)	0.66	0.61
	25–30	15/121	12.4%	1.45 (0.70 to 3.01)		1.49 (0.70 to 3.15)		
	≥30	8/75	10.7%	1.24 (0.52 to 2.96)		1.05 (0.42 to 2.60)		
	<25	29/220	17.5%	1.00 (ref.)	0.96	1.00 (ref.)	0.99	
Sacrocolpopexy	25–30	15/119	15.3%	1.00 (0.53 to 1.88)		0.91 (0.47 to 1.74)		
	≥30	5/47	13.5%	0.87 (0.33 to 2.25)		0.91 (0.34 to 2.38)		

BMI: body mass index (kg/m^2^).

**Table 3:** Comparisons between the three BMI groups according to the initial surgical approach of reoperation for prolapse recurrence, reoperation for SUI and for mesh-related complications.

	BMI (kg/m²)	Number of Events	Five-Year Rates	Hazard Ratio (95% CI)	*p*	Interaction *p*
Reoperation for Prolapse Recurrence						
	<25	16/382	4.8%	1.00 (ref.)	0.96	
All	25–30	10/240	4.8%	0.99 (0.44 to 2.18)		–
	≥30	6/122	5.7%	1.13 (0.44 to 2.89)		
Transvaginal mesh surgery	<25	6/162	3.7%	1.00 (ref.)	0.77	0.84
	25–30	4/121	3.3%	0.89 (0.25 to 3.14)		
	≥30	4/75	5.3%	1.43 (0.40 to 5.08)		
	<25	10/220	6.2%	1.00 (ref.)	0.95	
Sacrocolpopexy	25–30	6/119	6.8%	1.17 (0.42 to 3.21)		
	≥30	2/47	5.4%	1.00 (0.21 to 4.56)		
**Reoperation for SUI**						
	<25	22/382	6.5%	1.00 (ref.)	0.80	
All	25–30	11/240	4.9%	0.79 (0.38 to 1.64)		–
	≥30	6/122	5.5%	0.83 (0.33 to 2.05)		
Transvaginal mesh surgery	<25	8/162	4.9%	1.00 (ref.)	0.94	0.78
	25–30	6/121	5.0%	1.01 (0.35 to 2.91)		
	≥30	3/75	4.0%	0.81 (0.21 to 3.04)		
	<25	14/220	8.4%	1.00 (ref.)	0.73	
Sacrocolpopexy	25–30	5/119	4.4%	0.68 (0.24 to 1.88)		
	≥30	3/47	9.4%	1.06 (0.30 to 3.70)		
**Reoperation for mesh-related complications**						
	<25	8/382	2.3%	1.00 (ref.)	0.35	
Total	25–30	10/240	4.7%	1.98 (0.78 to 5.02)		–
	≥30	4/122	3.3%	1.56 (0.47 to 5.20)		
Transvaginal mesh surgery	<25	3/162	1.9%	1.00 (ref.)	0.54	0.91
	25–30	5/121	4.1%	2.23 (0.53 to 9.34)		
	≥30	2/75	2.7%	1.45 (0.24 to 8.68)		
	<25	5/220	2.9%	1.00 (ref.)	0.52	
Sacrocolpopexy	25–30	5/119	5.9%	1.90 (0.55 to 6.56)		
	≥30	2/47	4.6%	2.05 (0.39 to 10.59)		

BMI: body mass index (kg/m^2^).

## Data Availability

The data used to support the findings of this study are available from the corresponding author upon request.
